# The double helix model: Dynamic evolution of knowledge absorptive capacity and innovation efficiency

**DOI:** 10.1371/journal.pone.0336530

**Published:** 2025-11-20

**Authors:** Liangyou Cheng, Yong Qiu, Luwei Wang

**Affiliations:** 1 School of Digital Economy and Management, Sichuan Technology and Business University, Meishan, China; 2 College of Management, Sichuan Agricultural University, Chengdu, China; Southwestern University of Finance and Economics, CHINA

## Abstract

This study aims to propose a “double helix” dynamic evolution model of absorptive capacity and innovation efficiency, breaking through traditional linear cognition and revealing the synergistic growth patterns between the two. The research focuses on factors such as absorptive capacity, innovation efficiency, policy environment, and knowledge spillovers, using panel data from 29 countries spanning 1960–2023. By employing fixed-effects models, instrumental variable methods, and constructing composite indicators to measure core variables, the study analyzes the relationships through grouped regressions and robustness checks. The findings reveal a marginally enhancing convex positive effect of absorptive capacity on innovation efficiency. The policy environment strengthens this promoting effect by optimizing the institutional context. In high knowledge spillover environments, the convex relationship remains stable, while in low spillover environments, excessive absorptive capacity suppresses efficiency. Heterogeneity analysis shows that absorptive capacity plays a more significant role in the early stages of economic development and before 2000. Theoretically, this study improves the framework of innovation efficiency, and practically, it provides a basis for formulating precise innovation policies and dynamically adjusting innovation strategies for enterprises.

## Introduction

Innovation efficiency has become a core topic in global innovation research, essentially focusing on optimizing the relationship between innovation input and output conversion [1]. Innovation efficiency reflects the output effectiveness of R&D investment, encompassing the dynamic balance of knowledge flow and resource allocation [2]. In the era of digital economy, innovation efficiency directly impacts the effectiveness of enterprise digital transformation [3]. High innovation efficiency significantly enhances the likelihood of breakthroughs in green technologies [4] and promotes the optimization and upgrading of industrial structures [5]. The cross-country differences in innovation efficiency reflect the maturity of national innovation systems [6]. Under the context of open innovation, innovation efficiency determines the ability of enterprises to acquire external knowledge [7]. In emerging economies, improving innovation efficiency helps overcome resource constraints [8]. However, there are still gaps in the current understanding of innovation efficiency [9], which urgently requires the construction of a more systematic theoretical framework.

Existing views emphasize the decisive role of R&D investment in innovation efficiency [10], while neglecting the dynamic regulatory effect of absorptive capacity [11]. Some scholars have identified a U-shaped relationship between firm size and innovation efficiency [12], but they have failed to explain the micro-mechanisms behind this non-linear feature. In contrast to these studies, we propose a “double helix” evolutionary path for absorptive capacity, revealing its synergistic growth pattern with innovation efficiency [13]. Regarding policy influence, the existing literature predominantly focuses on the direct subsidy effects [14], while we find that the policy environment indirectly enhances innovation efficiency by reshaping knowledge networks [15]. Additionally, although some studies address the moderating mechanism of the policy environment on innovation efficiency, their analysis is not comprehensive enough [12,16]. Unlike existing research, we adopt a dynamic evolutionary perspective, considering the synergistic effects of absorptive capacity, policy environment, and knowledge spillovers. We construct a novel theoretical framework to more comprehensively and deeply reveal the mechanisms of innovation efficiency formation and enhancement [17,18].

Based on a systematic review of the existing literature, we propose the following core questions: Is there a more complex non-linear relationship, such as a marginally increasing convex relationship, between absorptive capacity and innovation efficiency? How can the policy environment enhance the promoting effect of absorptive capacity on innovation efficiency through optimizing institutional supply and resource allocation? How do different types and intensities of knowledge spillovers moderate the relationship between absorptive capacity and innovation efficiency in different contexts? To answer these questions, we use panel data from 29 countries spanning 1960–2023 [19,20] to construct a “double helix model” of innovation efficiency. Using fixed-effects models, instrumental variable methods, and other econometric techniques, we analyze the dynamic evolutionary path between absorptive capacity and innovation efficiency [21,22]. Through grouped regressions, we examine the heterogeneity of factors affecting innovation efficiency across different economic development levels, time periods, and knowledge spillover contexts, and ensure the reliability of our findings through a series of robustness checks [23,24].

Our marginal contributions are twofold. First, we innovatively propose the “double helix” dynamic evolution model of absorptive capacity and innovation efficiency, revealing the marginally enhancing convex relationship between them. This breakthrough challenges the limitations of traditional linear thinking and provides a new perspective for understanding the mechanisms of innovation efficiency improvement. Second, we construct a “capability-policy-spillover” synergistic system, systematically explaining how the policy environment, through institutional innovation and resource guidance, and knowledge spillovers, through external knowledge supply and sharing, jointly enhance the promoting effect of absorptive capacity on innovation efficiency. This offers a more scientific and comprehensive theoretical guide for the precise formulation of national innovation policies and the effective implementation of enterprise innovation strategies.

## Literature review and hypotheses

### Innovation efficiency

Innovation efficiency is a key indicator of the conversion of innovation inputs into outputs, reflecting an organization’s ability to achieve technological breakthroughs and market value under resource constraints [1]. Innovation efficiency encompasses the output effectiveness during the R&D phase and also involves resource allocation optimization in the commercialization process [25]. High innovation efficiency means that enterprises can achieve innovation goals at lower costs and faster speeds, gaining a competitive advantage [26]. Innovation efficiency includes the synergy between dynamic capabilities and static resources, and its essence lies in reducing friction losses during the innovation process through knowledge integration and process optimization [9]. Innovation efficiency has become a core element of national technological competitiveness and influences the speed and quality of industrial upgrading [27]. For emerging economies, improving innovation efficiency helps mitigate resource misallocation [8] and provides intrinsic momentum for sustainable development [4].

Innovation efficiency may be hindered by absorptive capacity and information barriers. On the one hand, insufficient internal absorptive capacity limits the effectiveness of external technology conversion [28], such as the diminishing marginal returns caused by the non-linear relationship between R&D investment and output [29]. On the other hand, information barriers in cross-departmental collaborations reduce the coherence of the innovation process [30], which is particularly evident in open innovation [31]. Previous studies have focused more on the static measurement of innovation efficiency, such as the ratio of patents to R&D expenditure [32], while neglecting its dynamic evolutionary characteristics [33]. Scholars tend to analyze the efficiency performance in a single technological domain [34] and rarely explore the synergistic effects of cross-technology integration [13].

### Absorptive capacity role in innovation efficiency

Innovation efficiency depends on a firm’s ability to recognize and integrate external knowledge [11]. Absorptive capacity optimizes economies of scale and scope in R&D by promoting an inverted U-shaped relationship between technological similarity and innovation performance [35], dynamically adjusting the boundary conditions for innovation efficiency [36]. When firms have high absorptive capacity, they can more efficiently absorb cross-national technology spillovers [37] and reduce R&D redundancy through open innovation [7], improving the conversion rate of innovation efficiency [38]. Furthermore, absorptive capacity mitigates the short-term negative impacts of digitalization on innovation efficiency [15], enhances technological assimilation efficiency in international mergers and acquisitions [36], and forms cumulative advantages in innovation efficiency [39]. This capability is manifested not only in the rapid application of explicit knowledge but also in the in-depth exploration of tacit knowledge [40], playing a leverage role in the long-term evolution of innovation efficiency [41].

The effect of absorptive capacity on innovation efficiency increases with the intensity of the capacity [12]. On the one hand, low levels of absorptive capacity may limit innovation efficiency due to bottlenecks in knowledge conversion [16], while moderate levels enhance innovation efficiency through collaborative R&D [17]. On the other hand, high absorptive capacity breaks through the path dependency of technological lock-in, achieving leaps in innovation efficiency through bidirectional knowledge flow [18] and integration into global value chains [42]. For example, in the field of green innovation, absorptive capacity regulates the interaction between core competition and environmental regulation [43], pushing innovation efficiency from incremental improvements to breakthrough changes [44]. This dynamic evolution follows a convex relationship of “capability-efficiency.” In the initial stage, absorptive capacity improves innovation efficiency slightly through basic knowledge accumulation [23]. In the middle stage, it accelerates innovation efficiency growth through the synergistic effects of organizational routines and external cooperation [24]. In the later stage, innovation efficiency undergoes an exponential breakthrough through knowledge recombination and disruptive learning [45]. Taken together, we predicted.

**H1** Absorptive capacity has a positive effect on innovation efficiency, and there exists a marginally enhancing convex relationship between the two.

### Policy’s moderating role between absorptive capacity and innovation efficiency

The improvement of innovation efficiency relies not only on a firm’s internal knowledge integration capacity but also on direct intervention and indirect guidance from external policy tools [46]. For example, government R&D subsidies and tax incentives strengthen a firm’s ability to convert external knowledge into innovation outputs by reducing innovation costs [14]. Regional open policies accelerate the accumulation of innovation efficiency through technology spillovers [47]. The precision of policy design is crucial, as targeted support for high absorptive capacity firms can avoid resource misallocation and maximize innovation efficiency [48]. Additionally, environmental regulation policies indirectly enhance innovation efficiency by forcing firms to optimize technological paths [5]. Intellectual property protection policies encourage original innovation by reducing imitation risks [49].

At the macro level, national innovation strategies coordinate regional innovation efficiency differences through top-level design [19], while industrial policies (e.g., high-tech industry support) guide firms’ absorptive capacity toward innovation efficiency conversion through technological standards [50]. At the meso level, cluster policies promote industry-academia-research cooperation, compensating for insufficient absorptive capacity of individual firms [51]. For example, in Norway’s energy efficiency innovation, government-led university-enterprise cooperation significantly improved innovation efficiency [52]. At the micro level, the combination effect of policy tools is key. For example, the simultaneous use of tax incentives and R&D subsidies can address short-term financial constraints and long-term capacity-building issues related to innovation efficiency. However, excessive policy intervention may suppress market selection mechanisms and reduce innovation efficiency [53]. Policies need to dynamically adapt to the stage of firms’ absorptive capacity [54].

When policy tools (e.g., technology transfer platforms) overlap with a firm’s knowledge base, they may enhance innovation efficiency [55]. However, policy lag or misalignment may lead to a disconnect between absorptive capacity and innovation efficiency [56]. For example, EU trade network policies enhance member states’ innovation efficiency through knowledge spillovers [57], while China’s mixed-ownership reform optimizes state-owned enterprise innovation efficiency through non-state shareholder governance [58]. Thus, we predicted.

**H2** Policy influences can enhance the promoting effect of absorptive capacity on innovation efficiency.

### Knowledge spillovers’ differentiated impact on absorptive capacity and innovation efficiency

Firms with high absorptive capacity can convert external knowledge into improvements in innovation efficiency, whereas firms with low absorptive capacity may experience a decline in innovation efficiency due to knowledge redundancy [59,60]. For example, in the global value chain, knowledge spillovers enhance innovation efficiency by accelerating technology diffusion, but over-reliance on external knowledge may suppress internal R&D efficiency [61,62]. The type of knowledge spillover (e.g., explicit vs. tacit knowledge) has different effects on innovation efficiency, with tacit knowledge requiring stronger absorptive capacity for conversion into innovation efficiency [63,64].

In high knowledge spillover environments, firms need to balance exploration and exploitation capabilities to maintain innovation efficiency [21,65]. For instance, social technology adoption improves innovation efficiency through rapid absorptive capacity, but failures in collective collaboration indicate that the social embeddedness of knowledge spillovers can weaken innovation efficiency [66]. In contrast, in low knowledge spillover environments, firms rely more on internal knowledge recombination to improve innovation efficiency, making the role of absorptive capacity more significant [67,68]. In high-tech opportunity environments, the marginal effect of knowledge spillovers on innovation efficiency decreases, whereas in low-tech opportunity environments, the critical role of absorptive capacity becomes more prominent [69,70].

Under high knowledge spillover conditions, absorptive capacity enhances innovation efficiency by reducing information asymmetry, but may form an inverted U-shaped relationship due to “complexity costs” [71,72]. In low knowledge spillover conditions, absorptive capacity drives innovation efficiency more directly through internal knowledge integration [73,74]. Thus, we predicted.

**H3** The level of knowledge spillovers moderates the relationship between absorptive capacity and innovation efficiency, with different mechanisms under high and low knowledge spillover conditions.

## Methods

### Research setting and data collection

Innovation efficiency is central to the study, and we base our analysis on the World Bank’s cross-national panel data from 1960 to 2023, focusing on 29 countries, including Albania, Algeria, and Afghanistan. These countries represent a broad spectrum of economic development, encompassing high, medium, and low-income nations, which allows for a comprehensive examination of the heterogeneous relationship between absorptive capacity and innovation efficiency [19]. Furthermore, these countries exhibit diversity in terms of technological innovation, knowledge spillovers, and policy environments, providing rich institutional contexts for testing moderation and threshold effects [20]. Additionally, the geographical distribution of the sample, covering Asia, Africa, Europe, and the Americas, helps mitigate regional bias, ensuring the robustness of the study’s findings [75]. To ensure data representativeness, we exclude countries with data missing for over 30% of the observations, and perform a Hausman test to verify the applicability of the fixed-effects model, controlling for unobserved country heterogeneity [76].

For variable construction, innovation efficiency (the dependent variable) is synthesized using two indicators: the proportion of high-tech exports and the number of articles in scientific journals. These indicators are standardized and log-transformed to eliminate dimensionality effects [58]. Absorptive capacity (the independent variable) is constructed by integrating three dimensions: technology import capacity, R&D capacity, and information technology infrastructure [77]. Control variables include traditional factors like economic size and urbanization level, while international knowledge spillovers and policy impacts are introduced as moderating variables to capture the external environment’s effects on the core relationship [21]. We use the instrumental variable approach to address endogeneity issues, confirming the validity of instruments via the Kleibergen-Paap test [22].

We employ a progressive analytical approach, beginning with descriptive statistics and correlation analysis to preliminarily explore variable relationships. The baseline regression model tests the non-linear impact of absorptive capacity and its square term on innovation efficiency, controlling for year and country fixed effects. We then introduce policy interaction terms to examine institutional moderation, including control variables such as economic size. The Hausman test confirms the use of the fixed-effects model, and we analyze heterogeneity by grouping countries according to knowledge spillover levels. Robustness checks include alternative variable methods, exclusion of outliers, and instrumental variable techniques, with the instrument’s validity confirmed through the weak instrument test (F = 77.86). Finally, we perform group regressions by economic development level and time period to examine the varying roles of absorptive capacity under different scenarios. Our final sample comprises 1,856 observations from 1960 to 2023.

### Variable selection

#### Dependent variable.

Innovation efficiency (*IE*) is measured using a composite indicator formed by the standardized high-tech export share (*IC*) and the number of articles in scientific journals (*KO*). The high-tech export share reflects the ability to convert technological outputs [53], while the number of scientific articles indicates the level of knowledge innovation output [36]. Together, these indicators comprehensively capture both the technological conversion and knowledge creation dimensions of innovation activities [47]. To ensure robustness, we take the logarithm of the number of journal articles to address skewness [78] and standardize all variables to remove dimensional differences [41]. The lagged innovation efficiency (*LIE*) is generated using a panel lag operator to control for dynamic effects [60]. This measurement method avoids the limitations of single indicators [12], aligns with the multidimensional concept of innovation efficiency [9], and better reflects sustained innovation potential compared to traditional indicators like patent counts [29].

#### Independent variables.

Absorptive capacity (*AC*) is constructed using multiple dimensions, integrating empirical findings related to technology import capacity, R&D capacity, and infrastructure [25,71]. Technology import capacity (*TIC*) is measured as the logarithm of royalty payments for intellectual property (BoP current US dollars), reflecting the economic investment organizations make to acquire external technological knowledge [1,56]. R&D capacity (*RDC*) is measured by the number of R&D personnel per million population, indicating the core human capital for internal knowledge conversion [34,67]. Information technology infrastructure (*ITI*) is evaluated by the number of fixed broadband subscriptions per 100 people, representing the material foundation for knowledge dissemination and integration [79,80]. These three dimensions correspond to the identification, assimilation, and application stages of absorptive capacity, and their arithmetic mean forms the final independent variable *AC*_*1*_. This method overcomes the bias of single indicators and resonates with [81]’s three-dimensional learning process of exploration-assimilation-development, while standardization eliminates dimensionality issues [32].

#### Control variables.

In addition to controlling for country and year fixed effects, we select seven key control variables to account for external factors that may affect innovation efficiency. Economic size controls for differences in national resource endowments, as previous studies show that the size of an economy directly affects the scale of R&D investment [10,49]. Economic development level captures the effects of development stage, as high-income countries tend to have better innovation infrastructure [51,70]. Market size reflects demand-pull effects, as larger markets can help absorb the fixed costs of innovation [31,61]. Education investment measures human capital accumulation, which directly influences knowledge assimilation capacity [23,68]. Urbanization represents the knowledge agglomeration effect, as urbanization provides the geographical platform for knowledge spillovers [6,82]. Labor market conditions control for human resource allocation efficiency, as employment pressure may suppress innovation investment [83,84]. Openness captures the effect of international knowledge flows, as more open economies are more likely to acquire external technologies [85,86].

First, economic size and development level form the material basis for a country’s innovation system [8,72]. Second, education investment and urbanization reflect the accumulation and distribution of knowledge production factors [45,73]. Finally, labor market conditions and openness regulate the institutional environment for innovation activities [63,74]. By controlling for these variables, we effectively isolate the macroeconomic environment’s influence on the core explanatory variables, ensuring that our estimation of the relationship between absorptive capacity and innovation efficiency is more accurate. As [16] point out, neglecting market size may overestimate absorptive capacity’s role, and [44] demonstrate collinearity between education investment and innovation efficiency. Through systematic control of these dimensions, we ensure a purer estimate of the relationship between absorptive capacity and innovation efficiency.

A detailed list of variables, operationalization, and reference is shown in **Table 1**.

**Table 1:** Variables, measures, and references.

Variables	Operational measure	Modeling Symbol	Reference
**Innovation efficiency**	New generated variable	*LIE*	[9,12,29,36,41,47,53,60,78]
**Absorptive capacity**	New generated variable	*AC*	[1,25,32,34,56,67,71,79–81]
**Economic size**	GDP in current local currency (LCU)	*ES*	[8,10,49,72]
**Economic development**	Per capita GDP in current LCU	*ED*	[8,51,70,72]
**Market size**	Total population	*MS*	[31,61]
**Education investment**	Government education expenditure as a percentage of GDP	*EI*	[23,45,68,73]
**Urbanization**	Proportion of urban population to total population	*UR*	[6,45,73,82]
**Labor market**	Percentage of youth (15–24 years) in total labor force	*LM*	[63,74,83,84]
**Openness**	Trade in goods as a percentage of GDP	*OP*	[63,74,85,86]
**Knowledge spillovers**	Foreign direct investment net inflow as a percentage of GDP	*KS*	[17,39,57,72]
**Technology Absorption**	Payments for intellectual property royalties, BoP in current US dollars	*TA*	[33,35,75,77]
**Technology transfer**	Exports of medium and high-tech manufactured goods	*TT*	[42,46,53,61]
**Basic education impact**	Duration of primary education	*BEI*	[55,72,84]
**Policy impact**	Government expenditure per student in higher education as a percentage of per capita GDP	*PI*	[38,57,82,87]

### Model specification

We use STATA 17.0 software to test our hypotheses. The core model is specified as follows:


$$LIE_{it}=\beta_{\text{0}}+\beta_{\text{1}}AC_{\text{it}}+\beta_{\text{2}}AC_{\text{it}}^{\text{2}}+\sum \beta_{\text{k}}{\text{X}}_{\text{kit}}+\alpha_{\text{i}}{+\gamma }_{\text{t}}{+\epsilon }_{\text{it}}$$ (1)

Where ${LIE}_{it}$ represents the lagged innovation efficiency of country i in year t, ${AC}_{it}^{\text{2}}$ is the squared term of absorptive capacity, used to examine its non-linear relationship with innovation efficiency, $X_{kit}$ is the set of control variables, including economic size (*ES*), development level (*ED*), etc., $\alpha_{i}$ and $\gamma_{t}$ are country fixed effects and time fixed effects, respectively, and $\varepsilon_{it}$ is the random error term.

## Results

### Main analysis

**Table 2** reports the descriptive statistics and Pearson correlations.

**Table 2:** Descriptive statistics and correlations.

Variables	Mean	S.D.	1	2	3	4	5	6	7
**1. Innovation efficiency**	0.00	0.72	1.000						
**2. Absorptive capacity**	0.09	0.57	0.145^***^	1.000					
**3. Economic size**	1.200 × 10¹²	6.530 × 10¹²	0.061^**^	0.023	1.000				
**4. Economic development**	6.92E + 04	2.52E + 05	0.036	0.027	0.607^***^	1.000			
**5. Market size**	4994423.00	1.120 × 10⁷	−0.029	−0.048	0.215^***^	0.006	1.000		
**6. Education investment**	5.56	8.87	0.077^***^	−0.014	−0.034	−0.059^**^	−0.082^***^	1.000	
**7. Urbanization**	55.74	25.25	0.189^***^	0.137^***^	0.060^**^	0.055^**^	−0.058^**^	0.003	1.000
	**Mean**	**S.D.**	**8**	**9**	**10**	**11**	**12**	**13**	**14**
**8. Labor market**	−1.35	169.19	1						
**9. Openness**	183.13	1270.88	−0.046^*^	1					
**10. Knowledge spillovers**	4.72E + 00	3.62E + 01	0.057^**^	−0.169^***^	1				
**11. Technology Absorption**	−3.640 × 10⁷	1.530 × 10⁹	0.037	0.037	0.01	1			
**12. Technology transfer**	19.50	22.75	−0.031	−0.004	−0.071^***^	−0.060^**^	1		
**13. Basic education impact**	5.55E + 00	1.20E + 00	0.333^***^	−0.270^***^	0.123^***^	0.103^***^	−0.190^***^	1	
**14. Policy impact**	1.19E + 02	2.85E + 02	0.027	−0.100^***^	0.027	−0.006	−0.092^***^	0.215^***^	1

Standard errors in parentheses.

*P < 0.1, **P < 0.05, ***P < 0.01.

In Model 1 of **Table 3**, the coefficient of *AC*_*1*_ is 0.266, and the coefficient of *AC*_*1*_^2^ is 0.230, both significant at the 1% level. In Model 2, these coefficients rise to 2.012 and 0.925, respectively, and remain significant at the 1% level. In Model 3, this structure remains stable with coefficients of 0.611 and 0.458. *AC*_*1*_^2^ is positive and significant in all models, indicating that the functional relationship curve between innovation efficiency and absorptive capacity is upward-opening, displaying a standard “convex” characteristic. In the regression equation, the marginal effect of absorptive capacity is determined by the derivative expression $\frac{dy}{dx}=\beta_{\text{1}}+\text{2}\beta_{\text{2}}X$. When $\beta_{\text{2}}$> 0, the marginal effect strengthens as absorptive capacity increases, meaning the higher the absorptive capacity, the stronger its effect on innovation efficiency. This increasing nonlinear incentivizing effect supports the nonlinear leap mechanism in the knowledge accumulation process emphasized by the “double helix model.” Therefore, H1 is validated.

**Table 3:** Main results.

	*LIE*			Model 4			Model 5	
	**Model 1**	**Model 2**	**Model 3**	**High knowledge spillover**	**Low knowledge spillover**	**Coefficient difference test**	**RE**	**FE**
***AC***_***1***_	0.266^***^ (0.092)	2.012^***^ (0.205)	0.611^***^ (0.134)	0.585^***^ (0.047)	0.434^**^ (0.173)	Δ = 0.151^*^	0.343^*^ (0.182)	0.611 (0.479)
***AC***_***1***_^**2**^	0.230^***^ (0.071)	0.925^***^ (0.099)	0.458^***^ (0.077)	0.273^***^ (0.034)	−0.586^**^ (0.289)	Δ = 0.859^***^	0.325^**^ (0.161)	0.458 (0.287)
***PI* × *AC***_***1***_		6.449^***^ (0.646)						
***PI***		0.111 (0.070)						
***ES***			0.663^***^ (0.075)	0.206^***^ (0.026)	0.364^***^ (0.068)	Δ = −0.158^**^	0.520^**^ (0.219)	0.663^*^ (0.285)
***ED***			−0.259^***^ (0.064)	−0.154^***^ (0.022)	−0.074 (0.051)	Δ = −0.080^*^	−0.251^*^ (0.151)	−0.259 (0.147)
***MS***			−1.025^***^ (0.371)	0.206^***^ (0.024)	−0.994^***^ (0.296)	Δ = 1.200^***^	−0.418^***^ (0.128)	−1.025 (0.842)
***EI***			0.130^***^ (0.049)	0.049^***^ (0.011)	0.166^***^ (0.025)	Δ = −0.117^***^	0.120 (0.109)	0.130 (0.095)
***UR***			0.291^**^ (0.128)	−0.136^***^ (0.016)	0.331^**^ (0.150)	Δ = −0.467^***^	−0.047 (0.074)	0.291 (0.495)
***LM***			0.328^**^ (0.138)	−0.041 (0.032)	−0.134^**^ (0.052)	Δ = 0.093^*^	0.262 (0.229)	0.328 (0.273)
***OP***			0.078^***^ (0.026)	−0.633^***^ (0.082)	0.075^***^ (0.008)	Δ = −0.708^***^	0.125^***^ (0.047)	0.078^*^ (0.038)
**Country**	Yes	Yes	Yes	–	–	–	–	–
**Year**	Yes	Yes	Yes	–	–	–	Yes	Yes
**R²(Within)**							0.287	0.297
**rho**							0.030	0.308
**_cons**	−1.448^***^ (0.207)	−1.084^***^ (0.240)	−0.781^***^ (0.253)	−0.145^***^ (0.022)	0.567^***^ (0.165)	Δ = −0.712^***^	−1.260 (1.292)	−0.715 (1.033)
**Adj-R** ^**2**^	0.159	0.385	0.297	0.659	0.277			
**F**	3.01^***^	6.14^***^	4.84^***^	43.58^***^	74.68^***^			
**N**	820	632	757	487	270		757	757

Standard errors in parentheses.

*P < 0.1, **P < 0.05, ***P < 0.01.

In Model 2 of **Table 3**, the coefficient of *PIAC* is 6.449, significant at the 1% level, indicating that the policy environment significantly enhances the positive impact of absorptive capacity on innovation efficiency. Policy investment does not directly drive innovation efficiency; instead, it amplifies the knowledge absorption potential of enterprises or regions by improving institutional conditions, exerting an indirect amplification effect. The main effect of *PI* itself is 0.111, which does not reach significance, further confirming its role as a moderating rather than a main effect variable. Comparing with Model 1, it is evident that after introducing the interaction term, the coefficients of *AC*_*1*_ and *AC*_*1*_^2^ rise from 0.266 and 0.230 to 2.012 and 0.925, respectively, indicating that the policy environment significantly amplifies the contribution of absorptive capacity to innovation efficiency at the margin. This mechanism aligns with the institutional dependency characteristic of absorptive capacity theory [87], emphasizing the role of external institutional foundations in capability transitions, thus confirming H2.

**Table 3** presents group regression analyses under high knowledge spillovers (Model 4) and low knowledge spillovers (Model 5). The results reveal structural differences in the mechanisms through which absorptive capacity operates in the two contexts. In the high knowledge spillover environment, the coefficients of *AC*_*1*_ and *AC*_*1*_^2^ are 0.585 and 0.273, respectively, both significant at the 1% level. This indicates that the impact of absorptive capacity on innovation efficiency continues to increase, with the marginal effect rising and the convex structure remaining stable.

In the low knowledge spillover environment, the coefficient of the first-order term is 0.434 (P < 0.05), still significantly positive, but the second-order term turns negative, with a coefficient of −0.586 (P < 0.05), reversing direction. This shows that after a certain level of absorptive capacity is reached, its effect on innovation efficiency becomes inhibitory, meaning the marginal effect decreases or even becomes negative. The coefficient difference test further confirms this heterogeneity: the difference in the second-order term Δ = 0.859 (P < 0.01) and the difference in the first-order term Δ = 0.151 (P < 0.1). This suggests that the knowledge spillover environment influences the effect of absorptive capacity, reshaping its marginal structure and reflecting the synergistic evolution mechanism between absorptive capacity and external knowledge supply. Therefore, H3 is supported.

### Robustness checks

#### Alternative variables.

First, in the alternative variable test, we replaced both innovation efficiency and absorptive capacity to validate the robustness of the conclusions drawn from the original hypothesis [87]. The results of different model analyses are presented in **Table 4**, where the coefficients remain significant and stable across models. The alternative indicator of innovation efficiency (Alternative LIE) was developed to capture the dynamic balance among innovation input, output, and conversion performance. Specifically, this composite measure integrates standardized indicators of patent applications by residents (representing innovation output), scientific publications (representing knowledge output), and the share of high-technology exports in manufactured exports (representing innovation conversion), while being adjusted by R&D investment intensity (representing innovation input). All component variables were standardized for cross-country comparability and combined using principal component analysis to mitigate dimensional and multicollinearity bias. In Model 1 and Model 2, the coefficient of *AC*_*1*_ increases from −0.833 to 6.534. Although the direction changes, it remains highly significant statistically (P < 0.01). This change indicates that the positive impact of absorptive capacity on innovation efficiency is universal, and whether using direct measures or alternative indicators, the significance of the results does not weaken due to the choice of variable. This further supports the conclusion in H1 that there is a positive relationship between absorptive capacity and innovation efficiency.

**Table 4:** Alternative variables.

	Alternative LIE			LIE	
	**Model 1**	**Model 2**	**Model 3**	**Model 4**	**Model 5**
***AC***_***1***_	−0.833^***^ (0.310)	6.534^***^ (0.671)	2.057^***^ (0.460)		
***AC***_***1***_^***2***^	0.161 (0.237)	3.200^***^ (0.330)	1.388^***^ (0.261)		
***PI × AC***		27.527^***^ (2.194)		0.777^***^ (0.287)	
***PI***		1.580^***^ (0.271)		−1.045^***^ (0.069)	
***AC***_***2***_				−1.266^***^ (0.123)	0.339^***^ (0.069)
***AC***_***2***_^**2**^				0.993^***^ (0.068)	0.613^***^ (0.069)
***ES***			1.762^***^ (0.261)		0.247^***^ (0.051)
***ED***			−0.758^***^ (0.230)		−0.250^***^ (0.039)
***MS***			−9.482^***^ (1.385)		1.197^***^ (0.245)
***EI***			0.294 (0.198)		0.115^***^ (0.029)
***UR***			3.468^***^ (0.433)		0.163^*^ (0.083)
***LM***			1.166^**^ (0.473)		0.301^***^ (0.045)
***OP***			−0.395^***^ (0.088)		−0.170 (0.153)
**Country**	Yes	Yes	Yes	Yes	Yes
**Year**	Yes	Yes	Yes	Yes	Yes
**_cons**	−6.394^***^ (0.708)	−5.187^***^ (0.800)	−2.234^**^ (0.918)	−2.419^***^ (0.215)	−0.290 (0.229)
**Adj-R** ^**2**^	0.150	0.431	0.308	0.558	0.454
**F**	2.74^***^	6.60^***^	4.73^***^	10.42^***^	12.06^***^
**N**	758	570	695	569	1198

Standard errors in parentheses.

*P < 0.1, **P < 0.05, ***P < 0.01.

Second, in the transition from Model 1 to Model 2, the coefficient of *AC*_*1*_^2^ rises from 0.161 to 3.200 and continues to remain highly significant. The nonlinear relationship between absorptive capacity and innovation efficiency still holds, and the convex relationship characteristic is evident under different alternative variables. As absorptive capacity increases, the effect of innovation efficiency improvement becomes more pronounced, and the increasing marginal effect remains unaffected by the use of alternative variables. Therefore, the use of alternative variables does not alter the marginal gain effect of absorptive capacity on innovation efficiency, further supporting H1 regarding the increasing incentivizing effect of knowledge absorption capacity. The enhancing effect of the policy environment on innovation efficiency is confirmed in Model 3 of **Table 4**. Under different alternative variables, the amplifying effect of policy impact on the relationship between absorptive capacity and innovation efficiency remains significant, indicating that the policy impact promotes innovation efficiency by improving the institutional context for absorptive capacity. The coefficient of *PI* × *AC* reaches 27.527 in Model 2 and remains statistically significant across different models. This suggests that the moderating effect of the policy environment positively reinforces the marginal effect of absorptive capacity, further validating H2, which posits that the policy environment enhances the positive impact of absorptive capacity on innovation efficiency.

Third, to further validate this finding, Models 4 and 5 introduce an alternative measure of absorptive capacity (AC_2_), which conceptualizes knowledge absorption from the perspectives of digital integration, information access, and human capital development. The AC_2_ composite indicator incorporates three standardized components, ITA (measured by ICT service exports as a share of total service exports), IC (measured by the proportion of individuals using the Internet), and HCB (measured by the share of highly educated labor within the working-age population). These variables jointly reflect a nation’s capacity to acquire, diffuse, and internalize technological knowledge through digital and human channels. The composite index was derived using principal component analysis, ensuring internal consistency and methodological comparability with AC_1_. In both Model 4 and Model 5, AC_2_ and its squared term remain statistically significant (p < 0.01), further confirming the robustness of the absorptive capacity–innovation efficiency nexus under different conceptualizations of absorptive capacity. Compared with Models 1–3, where AC_1_ and AC_1_^2^ consistently show positive and significant effects, the coefficients of AC_2_ (–1.266 and 0.339) and AC_2_^2^ (0.993 and 0.613) display similar significance patterns and maintain the same nonlinear convex structure. This cross-model consistency suggests that whether absorptive capacity is measured through R&D- and technology-import channels (AC_1_) or through ICT diffusion and human-capital dimensions (AC_2_), its positive and increasing marginal influence on innovation efficiency persists. Therefore, the results of the alternative variable test further consolidate the hypotheses proposed in the paper, proving the robustness of the conclusions.

#### Sensitivity and potential mechanism tests.

In **Table 5**, the coefficient of *AC*_*1*_ in Model 1 is 0.236 (P < 0.05). Although slightly lower than the baseline model, it still maintains a significant positive relationship. In Model 2, after introducing the square term, the coefficient of *AC*_*1*_^2^ rises to 0.993 (P < 0.01), making the convex curve characteristic more pronounced. This suggests that even when the precision of variable measurements changes, the marginal increasing effect of absorptive capacity on innovation efficiency remains valid. Furthermore, the coefficient of the policy moderation term, *PI* × *AC*_*1*_, reaches 6.952 (P < 0.01) in Model 2, further confirming that the strengthening effect of the policy environment on absorptive capacity is not altered by measurement adjustments. Therefore, the conclusions of H1 and H2 are robust.

**Table 5:** Sensitivity and potential mechanism analysis.

	Sensitivity test			Potential mechanism test	
	**Model 1**	**Model 2**	**Model 3**	**Model 4**	**Model 5**
***AC***_***1***_	0.236^**^ (0.099)	2.125^***^ (0.224)	0.456^***^ (0.148)	0.678^***^ (0.133)	1.184^***^ (0.244)
***AC***_***1***_^**2**^		0.993^***^ (0.110)	0.417^***^ (0.083)	0.470^***^ (0.076)	0.342^***^ (0.087)
***PI* × *AC***_***1***_		6.952^***^ (0.736)	0.238^***^ (0.076)		
***PI***		0.204^**^ (0.091)			
***KS***				0.394^***^ (0.086)	
***AC***_***1***_ *** × KS***				0.267^***^ (0.083)	
**ln*AC***					−0.462^***^ (0.165)
***AC***_***1***_ ** × ln*AC***					−0.547^*^ (0.297)
***ES***			0.680^***^ (0.084)	0.628^***^ (0.074)	0.689^***^ (0.075)
***ED***			−0.305^***^ (0.073)	−0.268^***^ (0.063)	−0.250^***^ (0.064)
***MS***			−0.238 (0.445)	−0.572 (0.379)	−1.224^***^ (0.376)
***EI***			0.277^***^ (0.063)	0.152^***^ (0.048)	0.159^***^ (0.050)
***UR***			0.154 (0.142)	0.455^***^ (0.131)	0.297^**^ (0.127)
***LM***			0.222 (0.151)	0.362^***^ (0.136)	0.429^***^ (0.142)
***OP***			0.053^*^ (0.029)	0.139^***^ (0.029)	0.092^***^ (0.026)
**Country**	Yes	Yes	Yes	Yes	Yes
**Year**	Yes	Yes	Yes	Yes	Yes
**_cons**	−1.624^***^ (0.235)	−1.303^***^ (0.283)	−1.330^***^ (0.305)	−1.034^***^ (0.256)	−0.924^***^ (0.257)
**Adj-R** ^**2**^	0.158	0.386	0.315	0.317	0.304
**F**	2.80^***^	5.51^***^	4.75^***^	5.17^***^	4.92^***^
**N**	741	553	678	757	757

Standard errors in parentheses.

*P < 0.1, **P < 0.05, ***P < 0.01.

Model 4 shows that the direct positive effect of knowledge spillovers on innovation efficiency is significant (coefficient 0.394, P < 0.01). The interaction term *AC*_*1*_ × *KS* has a coefficient of 0.267 (P < 0.01), indicating that an increase in knowledge spillover levels enhances the marginal contribution of absorptive capacity. This forms a logical closure with the conclusion from the group regression, where the effect of absorptive capacity is stronger in a high knowledge spillover environment. Additionally, Model 5, by introducing the logarithm-transformed absorptive capacity variable (ln*AC*) and its interaction term, finds that the coefficient of *AC*_*1*_ × ln*AC* is −0.547 (P < 0.1), suggesting that the marginal effect of absorptive capacity may exhibit a nonlinear turning point after reaching a certain threshold.

In the sensitivity and mechanism tests, the directions of the coefficients of control variables remain consistent with those in the baseline model. Economic size (*ES*) consistently shows a significant positive effect on innovation efficiency, while economic development level (*ED*) exhibits a suppressive effect. The significance fluctuations of variables like market size and education investment do not exceed the scope of the baseline model, indicating that the effects of core explanatory variables are not disturbed by adjustments to control variable measurements. Overall, the sensitivity and potential mechanism tests, from the perspectives of dynamic measurement, interaction effects, and control variables, further consolidate the robustness of the relationship between absorptive capacity and innovation efficiency, as well as the moderating effects of the policy environment and knowledge spillovers.

#### Endogeneity test.

To address potential endogeneity issues, we select technology transfer level (*TT*) as an instrumental variable for absorptive capacity. This variable is theoretically related to absorptive capacity through the knowledge acquisition and transformation path, as it influences the external knowledge processing processes of organizations, thereby affecting absorptive capacity. Moreover, it has a relatively weak direct relationship with innovation efficiency, meeting the requirements of both exogeneity and relevance for instrumental variables, and effectively mitigating reverse causality and omitted variable bias.

The results in **Table 6** show that under various methods, including IV estimation, 2SLS, LIML, and GMM, the coefficient of *AC*_*1*_ remains stable at 0.489 and highly significant (P < 0.01), consistent with the baseline model. This confirms that the positive effect of absorptive capacity on innovation efficiency is not driven by endogeneity. The impact of technology transfer level on absorptive capacity is significant (β = 0.221, P < 0.01), and the policy impact moderation effect is also significant (β = 0.326, P < 0.01), indicating that the core conclusions are not affected by endogeneity issues.

**Table 6:** Endogeneity test.

	IV estimation			2SLS		Robustness check	
	**Model 1** **IV (2SLS)**	**Model 2** **first_stage**	**Model 3** **second-stage**	**Model 4** **first_stage**	**Model 5** **second-stage**	**Model 6** **LIML**	**Model 7** **GMM**
***AC***_***1***_	0.489^***^ (0.047)		0.489^***^ (0.047)			0.489^***^ (0.047)	0.489^***^ (0.047)
***Instrumented AC***_***1***_					0.489^***^ (0.061)		
***TT***	0.221^***^ (0.016)	0.017 (0.027)	0.221^***^ (0.016)	0.017 (0.027)	0.221^***^ (0.021)	0.221^***^ (0.016)	0.221^***^ (0.016)
***KS***	0.020 (0.016)	0.003 (0.026)	0.020 (0.016)	0.003 (0.026)	0.020 (0.021)	0.020 (0.016)	0.020 (0.016)
***GO***	0.004 (0.016)	0.033 (0.027)	0.004 (0.016)	0.033 (0.027)	0.004 (0.021)	0.004 (0.016)	0.004 (0.016)
***PI***		0.326^***^ (0.036)		0.326^***^ (0.036)			
***BEI***		0.004 (0.052)		0.004 (0.052)			
***ES***	−0.018 (0.014)	0.018 (0.026)	−0.018 (0.014)	0.018 (0.026)	−0.018 (0.020)	−0.018 (0.014)	−0.018 (0.014)
***ED***	−0.017 (0.015)	−0.031 (0.026)	−0.017 (0.016)	−0.031 (0.026)	−0.017 (0.021)	−0.017 (0.015)	−0.017 (0.016)
***MS***	−0.009 (0.017)	0.010 (0.027)	−0.009 (0.017)	0.010 (0.027)	−0.009 (0.022)	−0.009 (0.017)	−0.009 (0.017)
***EI***	−0.014 (0.015)		−0.014 (0.015)	0.029 (0.027)	−0.014 (0.019)	−0.014 (0.015)	−0.014 (0.015)
***UR***	0.039^**^ (0.016)		0.039^**^ (0.016)	0.016 (0.027)	0.039^*^ (0.022)	0.039^**^ (0.016)	0.039^**^ (0.016)
***LM***	−0.036^**^ (0.015)		−0.036^**^ (0.015)	0.017 (0.027)	−0.036^*^ (0.020)	−0.036^**^ (0.015)	−0.036^**^ (0.015)
***OP***	0.020 (0.016)		0.020 (0.016)	0.059^**^ (0.025)	0.020 (0.021)	0.020 (0.016)	0.020 (0.016)
**Country**	Yes	Yes	Yes	Yes	Yes	Yes	Yes
**Year**	Yes	Yes	Yes	Yes	Yes	Yes	Yes
**e_hat**			0.021 (0.050)				
**_cons**	0.167^**^ (0.066)	−0.077 (0.113)	0.167^**^ (0.067)	−0.077 (0.113)	0.167^*^ (0.089)	0.167^**^ (0.066)	0.166^**^ (0.067)
**Adj-R²**	0.525	0.150	0.525	0.150	0.182	0.525	0.525
**F**	20.13^***^	8.76^***^	55.49^***^	8.76^***^	11.82^***^	20.13^***^	20.13^***^
**N**	1000	1000	1000	1000	1000	1000	1000

Standard errors in parentheses.

*P < 0.1, **P < 0.05, ***P < 0.01.

From **Table 7**, the Kleibergen-Paap rk LM statistic of 114.78 rejects the under-identification hypothesis, and both the Cragg-Donald Wald F statistic of 78.54 and the Kleibergen-Paap Wald rk F statistic of 77.86 are well above the critical value, ruling out the weak instrument issue. The Anderson-Rubin Wald test statistic of 32.27 and the Stock-Wright LM S statistic of 61.75^***^ indicate a strong correlation between the instrumental variable and the endogenous variable. The Hansen J over-identification test, with a p-value of 0.005, shows that the instrumental variable selection is reasonable, further confirming the robustness of the convex relationship between absorptive capacity and innovation efficiency and the moderating effect of policy.

**Table 7:** Endogeneity test validity analysis.

Test type	Statistic/indicator	Result
**Identification insufficiency test**	Kleibergen-Paap rk LM statistic	114.78^***^
**Weak instrument test**	Cragg-Donald Wald F statistic	78.54
	Kleibergen-Paap Wald rk F statistic	77.86
**Endogeneity between endogenous and instrumental variables**	f_stat	77.86^***^
**Weak instrument robust inference**	Anderson-Rubin Wald test	32.27^***^
	Anderson-Rubin Wald test	65.98^***^
	Stock-Wright LM S statistic	61.75^***^
**Adjusted R²**	Adj R²	0.150
	F	177.13^***^
	Abs_cap_hat	1^***^ (0.075)
**Wu-Hausman test**	F	0.17
	P	0.679
**Over-identification test**	Hansen J	0.005

### Heterogeneity analysis

#### Economic development heterogeneity analysis.

In **Table 8**, for the high economic development group (Model 1), the coefficient of the first-order term for absorptive capacity is 0.867, and the coefficient of the second-order term is −0.269, both significant at the 1% level. This indicates that the positive impact of absorptive capacity on innovation efficiency exhibits diminishing marginal returns, likely because in regions with high economic development, the knowledge absorption capacity tends to saturate, and additional investments in absorptive capacity yield limited innovation gains.

**Table 8:** Economic development heterogeneity analysis.

	Economic Development Level			Group coefficient difference test		
	**Model 1** **high**	**Model 2** **mid**	**Model 3** **low**	**High-low group**	**High-mid group**	**Mid-low group**
***AC***_***1***_	0.867^***^ (0.130)	−0.196^***^ (0.059)	5.887^***^ (1.000)	X² = 5.00, p = 0.025	X² = 35.36, p < 0.001	X² = 7.39, p = 0.0066
***AC***_***1***_^**2**^	−0.269^***^ (0.088)	0.005 (0.034)	1.210^***^ (0.322)	X² = 13.72, p < 0.001	X² = 6.57, p = 0.010	X² = 6.47, p = 0.011
***ES***	0.043 (0.034)	0.518^***^ (0.040)	−0.447 (0.280)	X² = 1.83, p = 0.176	X² = 11.66, p < 0.001	X² = 3.93, p = 0.047
***MS***	1.183^***^ (0.235)	−0.949^***^ (0.215)	−2.266^**^ (0.899)	X² = 18.67, p < 0.001	X² = 28.41, p < 0.001	X² = 1.96, p = 0.162
***EI***	0.010 (0.076)	0.159^***^ (0.023)	0.491^***^ (0.171)	X² = 2.15, p = 0.142	X² = 3.34, p = 0.068	X² = 2.75, p = 0.097
***UR***	−0.238^***^ (0.081)	−0.446^***^ (0.068)	2.154^***^ (0.287)	X² = 12.36, p < 0.001	X² = 3.38, p = 0.066	X² = 14.85, p < 0.001
***LM***	−0.023 (0.126)	−0.109 (0.096)	2.226*** (0.512)	X² = 6.47, p = 0.011	X² = 0.33, p = 0.566	X² = 7.26, p = 0.0071
***OP***	0.730 (1.006)	0.165^*^ (0.092)	0.101^**^ (0.040)	X² = 0.76, p = 0.384	X² = 0.48, p = 0.488	X² = 0.59, p = 0.442
**Country**	Yes	Yes	Yes			
**Year**	Yes	Yes	Yes			
**_cons**	3.976^***^ (0.755)	−4.802^***^ (0.801)	32.311^***^ (4.547)			
**Adj-R²**	0.956	0.885	0.394			
**F**	223.31^***^	94.32^***^	12.17^***^			
**N**	196	233	328			

Standard errors in parentheses.

*P < 0.1, **P < 0.05, ***P < 0.01.

In the middle development group (Model 2), the first-order term coefficient is not significant, while the second-order term coefficient is close to zero, suggesting a weakening nonlinear relationship between absorptive capacity and innovation efficiency. This may be due to the resource allocation efficiency and knowledge conversion bottlenecks associated with the moderate development stage. In the low development group (Model 3), the coefficients of both the first and second-order terms are 5.887 and 1.210, respectively, and are highly significant, showing a significant increasing marginal effect of absorptive capacity on innovation efficiency. This confirms the breakthrough role of absorptive capacity accumulation in driving innovation efficiency at the early stages of economic development.

The coefficient difference tests further indicate that the differences between the high and low economic development groups are significant at the 5% and 1% levels for the first and second-order terms, respectively. This reveals that the economic development stage, by altering the resource base for knowledge absorption and the conversion environment, restructures the marginal relationship between absorptive capacity and innovation efficiency. In other words, a certain level of economic development is necessary to unlock the innovation potential of absorptive capacity. This provides new empirical evidence for the environmental dependency of the convex relationship in H1 and validates the robustness of the core conclusion in different economic development contexts.

#### Analysis of temporal heterogeneity.

**Table 9** shows that the nonlinear relationship between absorptive capacity and innovation efficiency exhibits significant stage differences. Before 2000 (Model 1), the coefficient of the first-order term for absorptive capacity was 0.933 (P < 0.01), and the coefficient of the second-order term was 0.348 (P < 0.05), presenting a typical convex relationship. This indicates that in this stage, the marginal promoting effect of absorptive capacity accumulation on innovation efficiency increased, likely due to the scarcity of external knowledge supply in the early stages of the knowledge economy and the prominent marginal value of absorptive capacity.

**Table 9:** Analysis of temporal heterogeneity.

	*LIE*		
	**Model 1** **(Before 2000)**	**Model 2** **(After 2000)**	**Coefficient difference test**
***AC***_***1***_	0.933^***^ (0.288)	0.730^***^ (0.148)	X² = 0.28, p = 0.597
***AC***_***1***_^**2**^	0.348^**^ (0.145)	−0.184^*^ (0.097)	X² = 6.57, p = 0.010
***ES***	0.359^***^ (0.126)	0.028 (0.047)	X² = 5.69, p = 0.017
***ED***	−0.850^***^ (0.314)	0.012 (0.021)	X² = 9.28, p = 0.002
***MS***	−0.716 (0.733)	−0.093 (0.256)	X² = 1.48, p = 0.223
***EI***	0.046 (0.095)	0.322^***^ (0.066)	X² = 4.65, p = 0.031
***UR***	0.689^***^ (0.160)	0.129 (0.084)	X² = 3.05, p = 0.081
***LM***	0.185 (0.279)	0.429^***^ (0.137)	X² = 2.83, p = 0.092
***OP***	0.065^*^ (0.036)	0.803 (0.811)	X² = 2.46, p = 0.117
**Country**	Yes	Yes	
**Year**	Yes	Yes	
**_cons**	−0.336 (0.390)	−0.063 (0.082)	
**Adj-R²**	0.313	0.931	
**F**	11.63^***^	184.09^***^	
**N**	468	289	

Standard errors in parentheses.

*P < 0.1, **P < 0.05, ***P < 0.01.

After 2000 (Model 2), although the first-order term coefficient remained significantly positive, it dropped to 0.730, and the second-order term coefficient turned negative at −0.184 (P < 0.1), indicating the dissolution of the convex structure and a trend of diminishing marginal returns. This is consistent with the reality of the accelerating globalization of knowledge, the diversification of knowledge spillover channels, and the weakening of competitive advantages in absorptive capacity. The coefficient difference test shows that the difference in the second-order term is significant at the 1% level (X² = 6.57, p = 0.010), confirming the temporal dynamics of the relationship between absorptive capacity and innovation efficiency. Combined with the coefficient evolution of economic size and development level variables, it can be seen that the changes in the institutional environment for the flow of knowledge elements after 2000 have restructured the innovation gain mechanism of absorptive capacity. This provides new evidence for the spatial-temporal dependence of the convex relationship in H1 and verifies the robustness of the core conclusion from a temporal perspective.

## Discussion

### Theoretical implications

First, our unique contribution lies in discovering that the impact of absorptive capacity on innovation efficiency exhibits a marginally increasing “convex” pattern, rather than the traditional linear or diminishing marginal model [13,38]. This finding challenges the assumptions in existing studies (e.g., [52,88]) that absorptive capacity effects diminish with scale growth, suggesting the existence of a “critical threshold” for knowledge accumulation, beyond which the innovation returns from absorptive capacity accelerate. This nonlinear leap mechanism aligns closely with the “double helix” metaphor: knowledge absorption and innovation efficiency do not simply add up but evolve synergistically along a spiral upward path. Additionally, we further reveal the moderating role of the policy environment, finding that policy investments do not directly enhance innovation efficiency but amplify the marginal effects of absorptive capacity by optimizing the institutional context [15,28]. This finding fills the gap in existing research [42,89] regarding the indirect role of policy, providing a more nuanced theoretical basis for innovation policy design.

Second, in contrast to existing literature [3,90] that focuses on static analysis, our heterogeneity analysis reveals that the mechanism of absorptive capacity dynamically changes with knowledge spillover levels and economic development stages. In high-knowledge spillover environments, the marginal effect of absorptive capacity continues to increase, whereas, in low-knowledge spillover environments, its effect may diminish due to insufficient knowledge supply (see Table 3). This finding resonates with Peeters et al.‘s [91] view on the “adaptive configuration” of absorptive capacity, but further highlights the boundary conditions of its nonlinear effects. Moreover, we find that the innovation returns from absorptive capacity are higher during the early stages of economic development (see Table 8), which aligns with Howell’s [39] conclusions on the role of absorptive capacity in transitional economies and supplements the micro-mechanisms of dynamic threshold effects. Through time heterogeneity analysis (see Table 9), we reveal the reconstruction of absorptive capacity value due to knowledge globalization: after 2000, the marginal returns of absorptive capacity declined, indicating that its contribution to innovation is constrained by the increasing external knowledge mobility [4,92]. These findings integrate fragmented contextualized research [27,57] and provide a dynamic system framework for absorptive capacity theory.

Third, in contrast to single-perspective studies (e.g., [43] focusing on green innovation; [50] focusing on labor productivity), we find that absorptive capacity, external knowledge spillovers, and the policy environment form a “trinity” collaborative system (see **Table 5**). This finding goes beyond the traditional “capability-environment” dualism [30,93] and reveals the multi-level interactive mechanism for improving innovation efficiency. The policy environment enhances the marginal effects of absorptive capacity by reducing knowledge conversion costs [94], while knowledge spillovers enrich the knowledge base [62]. This systemic perspective provides an integrative framework for understanding the complex causes of innovation efficiency [40,54].

### Managerial implications

In contrast to existing studies [33,46] that focus on the linear effects of technology transfer or FDI, we find that the marginal contribution of knowledge absorptive capacity to innovation efficiency exhibits a “convex” increasing pattern. This suggests that companies need to reassess the strategic priority of knowledge accumulation. Once absorptive capacity surpasses the critical threshold, its innovation returns will accelerate. Companies should avoid short-term investments and instead focus on continuously building internal knowledge foundations to break through the capacity threshold [7,24]. We reveal that the policy environment amplifies the effect of absorptive capacity by reducing knowledge conversion costs [14], suggesting that companies should direct policy resources toward the knowledge digestion stage rather than simply expanding R&D scale. This stands in stark contrast to the traditional “policy-driven” innovation model [69]. Research shows that in low-knowledge spillover environments, absorptive capacity may become ineffective (see Table 3), indicating that multinational corporations need to adopt differentiated innovation network strategies: focusing on knowledge acquisition in high-knowledge spillover regions, while prioritizing the development of localized knowledge conversion systems in low-spillover regions [37,48].

From the perspective of dynamic capabilities, our heterogeneity analysis provides a practical framework for managers to respond to market uncertainty. The study finds that innovation returns from absorptive capacity are highest in the early stages of economic development (see **Table 8**), requiring emerging market companies to adopt a “capability-first” strategy, such as rapidly enhancing technological decoding capabilities through industry-academia collaborations (e.g., Industry 4.0 knowledge integration proposed by [65]), and avoiding blindly pursuing the scale of technology imports. For mature companies, the time heterogeneity analysis (see **Table 9**) indicates a diminishing marginal return of absorptive capacity after 2000, suggesting that companies should balance knowledge acquisition with internal transformation in open innovation. When external knowledge mobility is high, companies should strengthen their knowledge selection and recombination mechanisms [2], to avoid efficiency losses due to “knowledge overload.” We reveal that improving innovation efficiency requires the synergy of three elements: “policy-capability-environment.” Headquarters can maximize subsidiary innovation output by constructing a knowledge hub system [95], matching regional policy dividends with local knowledge spillover dynamics.

### Limitation and future research

Several limitations should be addressed in future research. First, our study primarily validates the nonlinear relationship based on macro-regional data and does not delve into the specific pathways of the knowledge conversion process at the micro-level of firms [64]. Future research could incorporate an organizational behavior perspective to explore the interaction mechanisms of different management levels in the formation of absorptive capacity [55]. Second, although the study identifies the moderating role of the policy environment, it does not differentiate the impact of various policy tools on innovation efficiency, which limits the precision of managerial recommendations. Future studies could examine the matching effects of policy mixes and absorptive capacity types. Third, we validate the boundary effects of the knowledge spillover environment, but we have not explored the role of industry-specific technological characteristics [5] in modulating absorptive capacity thresholds. Future research could combine the theory of technological complexity to deepen the heterogeneity analysis.
